# Motivators and Barriers to HPV Vaccination: A Qualitative Study of Underserved Women Attending Planned Parenthood

**DOI:** 10.3390/vaccines10071126

**Published:** 2022-07-15

**Authors:** Emilia J. Fields, Suellen Hopfer, Jennifer R. Warren, Rhonda BeLue, Joel Lebed, Michael L. Hecht

**Affiliations:** 1 Department of Health, Society, and Behavior, Program in Public Health, University of California-Irvine, Irvine, CA 92697, USA; fieldse@uci.edu; 2 Department of Communication, Women and Gender Studies, African and African American Studies, George Mason University, Fairfax, VA 22030, USA; jwarre20@gmu.edu; 3 Department of Public Health, University of Texas San Antonio, San Antonio, TX 78249, USA; rhonda.belue@utsa.edu; 4 Planned Parenthood Southeastern Pennsylvania, Philadelphia, PA 19107, USA; joel.lebed@ppsp.org; 5 REAL Prevention, LLC, Clifton, NJ 07013, USA; hechtpsu@gmail.com

**Keywords:** HPV vaccine, Planned Parenthood, federally qualified health centers (FQHC), Black women, Latina women

## Abstract

Human papilloma virus (HPV) is the most common sexually transmitted infection in the United States. Disease-associated strains of HPV can cause genital warts and six cancer types. HPV-associated cervical cancer disproportionately impacts medically underserved women including Black and Latina women with respect to incidence, prevalence, and mortality rates. Although safe and effective vaccines are available, HPV vaccination rates remain low among low-income individuals and women of color. The current study examined individual and structural motivators and barriers to HPV vaccination among medically underserved women utilizing a Planned Parenthood health center in Southeast Pennsylvania. Guided by narrative engagement theory (NET), qualitative interviews (N = 24) were used to elicit HPV vaccine decision stories from both vaccinated and unvaccinated women. Using a phronetic iterative data analysis approach, we identified three motivators to vaccinate against HPV: (1) receiving an explicit vaccine recommendation from a healthcare provider (a structural determinant), (2) feeling empowered to take control of one’s health (an individual determinant), and (3) knowing someone infected with HPV (an individual determinant). Among unvaccinated participants, barriers to HPV vaccination included: (1) not receiving an explicit vaccine recommendation from a healthcare provider (a structural determinant), (2) low perceived risk for acquiring HPV or that HPV is not severe (an individual determinant), and (3) lack of maternal support to vaccinate (a structural determinant). Healthcare providers are optimally positioned to fill the gap in prior missed vaccine opportunities and empower women by recommending HPV vaccination.

## 1. Introduction

Human papilloma virus (HPV) is the most common sexually transmitted infection in the United States [1]. Every year, nearly 20,000 women and 12,000 men experience cancers caused by HPV [2]. Although most infections are self-limiting, HPV infection can lead to cervical, vaginal, vulvar, anal, and oropharyngeal cancers as well as genital warts in women [2,3,4,5]. In the U.S., HPV is responsible for approximately 13,000 new cases of cervical cancer and 4000 related deaths annually [6,7].

Cervical cancer disproportionately impacts medically underserved women [8,9]. For example, low-income and racial/ethnic minority women have higher prevalence, incidence, and cervical cancer mortality rates [8,10,11,12,13]. In the U.S., a multi-dose, nonavalent HPV vaccine (Gardasil-9) is available and offered to individuals age 9 to 45 years old [14,15]. The vaccine is safe and effective in protecting against the nine most common types of HPV that contribute to cancer and genital warts (HPV types 6, 11, 16, 18, 31, 33, 45, 52, and 58) [16,17,18,19]. Despite its safety and effectiveness, HPV vaccination among medically underserved adult women is low [20]. National data published in 2020 from 2013 to 2018 suggest that only 53% of women ages 18 to 26 were vaccinated with at least one dose [21]. Additionally, compared to white women (~58%), Latinas (~49%) and Black women (~45%) were less likely to receive one or more doses of the HPV vaccine [21]. Studies report that low-income and racial/ethnic minority women also have lower rates of completing the HPV vaccine series [22,23,24,25]. HPV vaccination disparities contribute to the observed disparities in cervical cancer [12]. National Cancer Institute (NCI)-designated cancer centers have called for an urgency in increasing HPV vaccination rates, which dropped by an estimated 20 percent during the COVID-19 pandemic [26].

To improve HPV vaccine uptake among medically underserved women attending safety net health centers, it is critical to understand key motivators and barriers unique to this population to be able to tailor vaccine messages and increase sub-optimal HPV vaccination [27]. Healthcare providers, especially those who see young adult women for preventive reproductive care at safety net health centers such as Planned Parenthood, may benefit from knowing which factors motivate or impede HPV vaccination. Previous studies examining motivators and barriers have generally focused on parent populations [28,29,30]. However, the Advisory Committee on Immunization Practices recommends that individuals 9 to 26 years old vaccinate against HPV (with shared clinical decision-making recommendations for 27- to 45-year-olds). As a result, it is important to reach adults who may not have had the opportunity to vaccinate as adolescents. Although studies have been conducted among young women attending college [31,32,33,34,35], fewer studies have focused on medically underserved women utilizing safety net health centers [25,36,37]. Given that safety net health centers traditionally serve women disproportionately impacted by HPV, additional research is needed to understand the HPV vaccine decision-making process among this population and identify communication and prevention opportunities.

The following study aimed to identify multi-level factors that reflect broader individual and structural motivators and barriers to HPV vaccination among medically underserved women utilizing the Planned Parenthood of Southeast Pennsylvania (PPSP) safety net health center for their healthcare needs. The goal of interviewing women attending PPSP was also to inform the adaptation of a National Cancer Institute (NCI) evidence-based cancer control program (EBCCP) [38]. The original video-based intervention was grounded in the HPV vaccine decision stories of college women [39]. With the goal of adapting the intervention to reflect decision stories from medically underserved women, we intentionally collaborated with Planned Parenthood. One of the leading safety net health centers in the U.S. [40], Planned Parenthood aims to reach women who may have less access to routine preventive care. Planned Parenthood centers are often physically situated in communities that are underserved and under-resourced [41]. In the adapted intervention, decision stories were meant to be delivered on a health kiosk placed in the waiting rooms of health centers.

## 2. Materials and Methods

The study involved conducting in-depth interviews (N = 24) with young adult women attending a PPSP health center located in a suburb of Philadelphia, PA, USA, in the summer months between 18 June to 16 July 2015. Women attending the PPSP health center were approached by one of three interviewers after checking into their medical appointment. Interviewers were similar in race/ethnicity, age, and gender to assist women in feeling comfortable. Interviewers explained the purpose of the study to women waiting in the waiting room and screened those who were interested in participating. The screening process consisted of asking women to complete a two-item questionnaire that inquired about their age and HPV vaccination status. The study’s eligibility requirements were as follows: (1) women aged 18 years old and older and (2) women who knew their HPV vaccination status, whether fully, partially, or un-vaccinated. Women who were fully or partially vaccinated against HPV were regarded as vaccinated participants. Eligible women were purposively enrolled into the study after reviewing the study information sheet and providing written informed consent. One-time on-site interviews were conducted in a private office located in the PPSP health center while participants waited to be called for their medical appointment. On average, interviews lasted approximately 30 to 60 min. Sociodemographic information was collected with a brief survey prior to the interview. Participants were emailed a twenty-dollar cash card immediately after completing the study.

### 2.1. Interview Guide

The development of the interview guide was informed by narrative engagement theory (NET) [42,43] to uncover motivators and barriers to HPV vaccination. The NET framework was used, as it enlists stories of the intended audience for the goal of developing and adapting preventative interventions. Two interview guides were developed for the current study. The interview guide for vaccinated participants asked women to share and reflect their HPV vaccination experience to uncover what factors, conversations, and contexts led them to vaccinate. The interview guide for unvaccinated women focused on discovering barriers to vaccination. Both interview guides used probes and prompts to follow up on vague responses to ensure the collection of rich data [44].

### 2.2. Data Analysis and Rigor

Audio recordings of the interviews were transcribed verbatim for accuracy, and women’s names were replaced with participant ID numbers. Four researchers (E.J.F., S.H., M.L.H., and J.R.W.) first read all transcripts, immersing themselves in the data (i.e., becoming familiar with the content of the data and what was being talked about). Subsequently, for data analysis, two researchers (E.J.F. and S.H.) engaged in the coding and interpretation process. A phronetic iterative approach to data analysis was implemented to prioritize praxis in the context of a safety net health center. Phronetic refers to the Greek term phronesis, meaning practical knowledge [45]. An iterative approach involved first inductively coding data with descriptive codes (primary level data analysis) to describe what women shared (i.e., the who, what where, when and how). Coders then deductively analyzed and organized data for multi-level motivators and barriers to HPV vaccination (secondary level data analysis) [46] from a socio-ecological perspective. A health disparities socio-ecological framework that describes multiple levels of influence across various domains and the lifespan was used to guide, contextualize, and organize descriptive codes into themes of motivators and barriers operating at different levels [47,48]. A codebook was developed in Microsoft Excel to reflect this data analysis process. Identified motivators and barriers were organized into individual or structural determinants of HPV vaccination. We defined structural determinants to be factors operating outside of the individual-level that influenced a participant’s vaccination decision or behavior [49,50]. Lastly, descriptive statistics were used to analyze participant’s sociodemographic data.

To enhance trustworthiness of qualitative data analysis, dependability was met with an inquiry audit trail consisting of verbatim transcripts for accuracy and a codebook documenting the data interpretation process. The codebook is available up request. Transferability of data was met with thick description of women’s decision stories. Credibility was met through, (1) site observations over a one-month period, (2) triangulation of methods including discussions with healthcare providers at the site about the prevalence of patients seen with HPV, and (3) peer debriefing among the authors and the site medical director (co-author J.L.) on how findings at this safety net health center may compare with other health centers in the area.

## 3. Results

Table 1 describes participant’s sociodemographic characteristics. Among the 24 women in the sample, the median age was 22, with ages ranging from 18 to 34 years. Women self-identified as African American/Black (N = 18), Latina (N = 2), Caucasian (N = 2), and mixed race/ethnicity (N = 2). Among the participants who identified as mixed race/ethnicity, one woman identified as Haitian, Hawaiian, and American Indian, and the other identified as White and Arab. Unvaccinated women comprised 38% (N = 9) of the sample. Of the fifteen vaccinated women, a majority 73% (N = 11) had completed the three dose HPV vaccine series, and nearly half were vaccinated as adolescents. While two doses are now recommended for adolescents under 15 years old starting the HPV vaccine series [14], three doses were recommended for all vaccine-eligible individuals at the time of the study. A majority of the sample reported being sexually active, not using condoms during sex, and having health insurance at the time of the interview. Lastly, one participant disclosed previously testing positive for HPV (genital warts).

The phronetic iterative data analysis approach identified individual and structural motivators and barriers to HPV vaccination. Motivators to vaccinate against HPV included: (1) receiving an explicit vaccine recommendation from a healthcare provider, (2) feeling empowered to take control of one’s health, and (3) knowing someone infected with HPV. Barriers to HPV vaccination included: (1) not receiving an explicit vaccine recommendation from a healthcare provider, (2) low perceived risk for acquiring HPV or that HPV is not severe, and (3) a lack of maternal support to vaccinate.

### 3.1. Motivators for Vaccination

#### 3.1.1. Receiving an Explicit Vaccine Recommendation from a Healthcare Provider

Among vaccinated women, receiving a vaccine recommendation from their healthcare provider emerged as a dominant structural motivator to vaccinate against HPV. Women vaccinated as adolescents recalled a healthcare provider recommending the HPV vaccine to their mother. Overall, recommendations were initiated by a variety of providers such as pediatricians, family practitioners, obstetricians, and gynecologists. The initial purpose of the medical appointment that led to vaccination included annual health check-ups, sexual health visits, needing a meningitis vaccine for college, and a prenatal check-up. Women cited phone calls, emails, and text message reminders from their provider’s office as cues to vaccinate for subsequent doses after initiating the series.

Women discussed their healthcare provider’s rationale for vaccinating against HPV. Those who vaccinated as adults recalled their providers citing becoming sexually active, starting college, an incomplete vaccination history, postpartum care, having an underlying health condition, and having a history of sexually transmitted infections as reasons to vaccinate. One woman shared, *“She [healthcare provider] told me since I’m going to be sexually active with my partner then it would be a good idea for me to get it [HPV vaccine]…”*—Vaccinated, African American, age 20. One participant discussed having an underlying health condition as another reason cited by her provider: *“I’m HIV positive and with me having HIV, they [healthcare provider] wanted to protect me from anything possible.”*—Vaccinated, African American, age 22. Additionally, after testing positive for HPV, another woman reported that her primary care physician recommended she vaccinate. 

Women who were vaccinated at a younger age were unable to recall the rationale provided to their mothers. However, one woman emphasized the likely role of trust in her mother’s decision: *“We’ve been at my doctor’s office ever since I was a baby. She’s been there forever. She trusts the opinion of the doctor.”*—Vaccinated, African American, age 22.

The HPV vaccine’s benefits were framed in various ways by healthcare providers. Participants recalled providers highlighting the vaccine’s ability to prevent cervical cancer. None of the providers reportedly discussed the vaccine as also preventing anal, oropharyngeal, or vaginal cancer. Nor did women report providers discussing the risk of acquiring HPV from oral sex. Additionally, women reported that only a few providers discussed the vaccine as protecting against genital warts. When asked about a preference for framing, one participant discussed the need to highlight protection from genital warts., *“I probably wouldn’t focus on cancer as much as Gardasil does. I would focus on protecting myself from genital warts, from certain strains because at that young population, you’re not thinking about cancer.”*—Vaccinated, African American, age 31.

Prior to vaccination, most women described having limited awareness about HPV and/or the vaccine. Some women contributed prior vaccine awareness to television commercials, health center posters, conversations at school, and discussions with friends, family, or co-workers. However, overall, many women reported learning about the vaccine through recommendations and conversations with their healthcare provider: *“I didn’t see any commercials, any advertisement. I just had went to the doctors and they told me about it.”*—Vaccinated, African American, age 20.

Women reported mixed experiences making an informed decision to vaccinate against HPV. While some women reported feeling informed, others discussed not receiving enough information or information in the desired format (e.g., written vs. verbal). This often resulted in women passively accepting the vaccine. One participant shared, *“Basically what happened was I went to the doctor. They looked at my shot history and they said this is what you need. They didn’t give no [pauses], a lot of details. They just was like here, these are the shots that you need.”*—Vaccinated, African American, age 23. For some women, a lack of information contributed to medical mistrust: *“…when me and my friends engage in discussions, we’re always saying we don’t know what that [vaccine] does, we don’t know the outcome. They treat us like guinea pigs.”*—Vaccinated, African American, age 23.

Women who had vaccinated as minors reported not being included in the decision-making process. Often, these women described a lack of autonomy and feeling forced into accepting the vaccine. One woman said, *“My mom pushed me to do it after my doctor mentioned it.”*—Vaccinated, African American, age 22. Another woman who was vaccinated with her sister as an adolescent echoed this sentiment by sharing, *“I was just along for the ride for the most part.”*—Vaccinated, Caucasian, age 20. As a result of feeling excluded, one 21-year-old Latina woman reported resenting her mother and declining to finish the series upon turning 18 years old. However, other women reported not caring or wanting to be included in the vaccine decision making process as minors. Interestingly, at the time of the interview, one woman who expressed this sentiment was unaware of what the HPV vaccine protected her against.

#### 3.1.2. Feeling Empowered to Take Control of One’s Health

Women reported feeling empowered by vaccinating and viewed vaccinating as one way to take charge of their health. This theme emerged as an individual motivator influencing some participant’s decision to vaccinate. For those who were sexually active, providers discussed the possibility of becoming infected by asymptomatic male partners. As a result, women talked about not wanting to rely on a partner for protection against HPV. One woman shared, *“I decided, yeah that’s a good idea because I don’t know what he [boyfriend] was doing in his past relationships, so I wanted to get it.”*—Vaccinated, African American, age 20. Women also shared how wanting to stay healthy played a role in their decision to receive the vaccine: *“If it’s going to assure or at least help me to stay healthy as much as possible, I get it.”*—Vaccinated, African American, age 25. Women with co-morbidities such as HIV went on to reiterate the importance of protecting oneself: *“I wanted to be on the safe side and protect myself from HPV.”*—Vaccinated, African American, age 22, HIV positive. Changes in one’s social environment also prompted women to vaccinate and protect themselves against HPV. One college bound woman shared, *“[It’s a] new environment, new people, better now than later. [It’s a] good thing to get just in case.”*—Vaccinated, African American, age 18.

In discussing how to motivate other women to vaccinate, participants echoed feelings of empowerment. Participants explicitly discussed taking charge of their sexual and reproductive health by vaccinating. One woman shared, *“It’s better safe than sorry and it’s better to be on point with your health because it’s very important especially as a female and especially if you plan on giving birth.”*—Vaccinated, mixed race/ethnicity, age 19. Another participant said, *“People don’t tell you all the things that can go wrong with your female reproductive system like infertility, breast, cervical, and ovarian cancer. Why would you not take advantage of this one preventative measure that’s super easy to do that can seriously lower your risk of one of these complications? Why is it that we don’t want to take care of our female reproductive organs?”*—Vaccinated, Caucasian, age 20. Additionally, one woman who was HPV positive with genital warts said, *“If you want to stay healthy then you should vaccinate, there’s no argument about it. You don’t want to go through this [genital warts]. If you care about your health, this is something you should do.”*—Vaccinated, mixed race/ethnicity, age 19.

#### 3.1.3. Knowing Someone Infected with HPV

Women discussed knowing family and/or friends infected with HPV as an individual motivator playing a significant role in their decision to vaccinate. One woman recalled a family friend as her mother’s motivation to vaccinate her (the interviewee) as an adolescent: *“I know her friend was diagnosed with it [cervical cancer]. That’s what made her want to get her child vaccinated.”*—Vaccinated, African American, age 22. Knowing someone with HPV was cited as a motivator to learn more about the vaccine. For example, one woman explained that knowing a pregnant aunt with HPV scared her into wanting to learn more about the HPV vaccine. Among participants, knowing someone with cervical cancer was a greater motivator to vaccinate than knowing someone with genital warts. Despite knowing friends with genital warts who self-described the disease as “severe”, some participants did not perceive it to be serious. One woman stated, *“In my opinion, my girlfriend’s situation [genital warts] is not all that bad. Not like women having to do chemo”*—Vaccinated, African American, age 25. Another participant minimized her friend’s HPV diagnosis by saying, *“I think I’ve had a friend who had HPV, but it wasn’t really anything big.”*—Vaccinated, Latina, age 21.

### 3.2. Barriers to Vaccination

#### 3.2.1. Not Receiving an Explicit Vaccine Recommendation from a Healthcare Provider

Among unvaccinated women, not receiving a healthcare provider recommendation was reported as a structural barrier to receiving the HPV vaccine. Approximately half of unvaccinated participants reported not receiving a recommendation to initiate the HPV vaccine series. After learning more about HPV from the interviewer, one woman said, *“So shouldn’t my doctor have recommended it?”* and *“I feel like my doctor should have said something about it.”*—Unvaccinated, Caucasian, age 18. Although some unvaccinated women reported hearing about the HPV vaccine from T.V. commercials and the Internet, others reported no prior awareness. Women highlighted the burden placed on patients when vaccine recommendations are not made, *“They [healthcare provider] never asked did I want to get it. They ask, ‘why you coming here?’ If I wanted it I guess I would have to tell them I want it.”*—Unvaccinated, African American, age 19. Despite not receiving a recommendation, participants discussed a willingness to vaccinate. When asked if she would vaccinate to protect against cervical cancer and genital warts, one woman said, *“I’m definitely with that, I would get vaccinated.”*—Unvaccinated, African American, age 19. One woman expressed needing maternal approval to vaccinate: *“I’ll have to ask my mom about it [HPV vaccine] first.”*—Unvaccinated, Caucasian, age 18.

#### 3.2.2. Low Perceived Risk for Acquiring HPV or That HPV Is Not Severe

Not feeling at risk for HPV was cited as an individual factor that led some women to refuse vaccination. This perception was reported among unvaccinated women who received a recommendation and information about the HPV vaccine. A few women did not perceive the vaccine as necessary because they were not sexually active when a recommendation was given: *“I wasn’t sexually active, so I just didn’t think I needed it.”*—Unvaccinated, African American, age 24. Sexually active women also reported not feeling at risk. For these women, HPV prevention was not perceived as a priority, life threatening or urgent. As a result, getting vaccinated was a low priority. For example, one woman who refused to continue the HPV series upon turning 18 years old shared, *“I’m not opposed to it anymore, as much as I was before but um, it’s definitely not high on my priority list although it should be.”* She further elaborated by saying: *“I know that it could lead to cervical cancer, but for the most part in most women, it doesn’t. It’s not like, if I thought it was more life threatening, need to do thing I would have more of a sense of urgency about it. It’s just like, everyone has it. A lot of women get it at some point.”*—Vaccinated, Latina, age 21.

#### 3.2.3. Lack of Maternal Support

Some women reported a lack of maternal support as another structural barrier to HPV vaccination. For example, one participant recalled that despite her willingness to vaccinate and her mother receiving a vaccine recommendation from a healthcare provider, her mother refused to vaccinate her as an adolescent. This woman went on to share that her mother worked as a nurse. During the interview, the participant expressed feeling confused by her mother’s decision to not vaccinate her: *“My mom is a nurse, that’s weird and she said no.”*—Unvaccinated, African American, age 25.

## 4. Discussion

The current study identified various individual and structural motivators and barriers to HPV vaccination among medically underserved women utilizing the PPSP safety net health center. Motivators to vaccinate against HPV were cited as: (1) receiving an explicit vaccine recommendation from a healthcare provider, (2) feeling empowered to take control of one’s health, and (3) knowing someone infected with HPV. Barriers to HPV vaccination included: (1) not receiving an explicit vaccine recommendation from a healthcare provider, (2) low perceived risk for acquiring HPV or that HPV is not severe, and (3) a lack of maternal support to vaccinate.

Receiving a recommendation from a healthcare provider was cited as the primary motivator for uptake of the HPV vaccine. This theme emerged among participants vaccinated as adults and those vaccinated as adolescents by their mothers. This finding is consistent with a large body of literature reporting provider recommendations as a strong predictor of HPV vaccination [51,52,53]. What is noteworthy is the importance of healthcare providers seeing adult women to inquire about their patient’s HPV vaccination status during medical appointments. Although pediatric healthcare providers may have recommended the HPV vaccine to women as adolescents, their parents may have declined vaccination. Therefore, for these adult women, asking about and recommending the HPV vaccine ensures that those who missed vaccination have the opportunity to catch up on beneficial HPV-associated cancer prevention. Similar to reported research, participants vaccinated as adolescents were often passive participants in the vaccine decision-making process [54]. Study findings suggest that a lack of participation may impede series completion and reduce knowledge about the HPV vaccine. With respect to adolescents, researchers have called for greater autonomy in the decision making process [55]. Healthcare providers should ensure vaccine-eligible children receive age-appropriate vaccine information and recommendations.

Women discussed vaccination against HPV as a way to gain control over their health status. A 2017 study conducted among women utilizing a safety net health center for preventative care reported a similar theme labeled as “independence” [36]. In the current study, empowerment was explicitly rooted in a desire to protect one’s sexual and reproductive health. Additionally, women expressed uncertainty about their partner’s HPV status and subsequently were less willing to rely on them for protecting their own reproductive health. Given the intersect of gender, race, and class, medically underserved women may experience powerlessness on multiple levels [56]. Therefore, encouraging self-responsibility as a form of sexual and reproductive empowerment may be beneficial. Previous research has found that incorporating empowerment into risk messaging is a persuasive message strategy [33]. Empowerment can also be achieved by ensuring that patients feel heard, respected, and informed when making vaccine decisions with their healthcare provider [55,57]. This point may be especially salient for populations that have historically and systemically encountered racism and discrimination.

Among study participants, low HPV risk perceptions were associated with misconceptions around disease severity and the vaccine’s utility. Women’s reproductive health will benefit from healthcare providers, including gynecologists, delivering high-quality HPV vaccine recommendations that are strong, educating, and express the urgency to vaccinate [58]. Providers should inquire about prior vaccine awareness and knowledge as well as desired learning formats when educating patients. Furthermore, providers should stress that vaccinating before sexual debut improves protection against HPV [59]. The study also suggests that risk perceptions may vary by disease type, with genital warts perceived as less severe than cervical cancer. Although genital warts is associated with less morbidity and mortality compared to HPV-related cancers, providers should highlight the psychological distress frequently associated with acquiring the virus, including genital warts and future partner interactions [60]. Providers can share relational focused HPV vaccine messages as a strategy to prioritize HPV vaccination.

Similar to prior research findings, knowing someone infected with HPV was associated with vaccine uptake [61]. Knowing someone with HPV may have implications for risk perceptions and awareness [61]. The current study adds that it may also contribute to increased HPV vaccine information seeking behaviors.

In our study, women who did not receive an HPV vaccine recommendation from their healthcare provider expressed a willingness to vaccinate. This suggests that unvaccinated women were not necessarily hesitant but were unaware of HPV and/or the vaccine in many cases and had not received a recommendation from a healthcare provider. The absence of an HPV vaccine recommendation highlights a missed opportunity to vaccinate those who are eligible. Furthermore, providers who do not issue recommendations place an unfair burden on patients who may be unaware of the vaccine and the potentially adverse health consequences of HPV.

The literature indicates that medically underserved women are less likely to receive HPV vaccine recommendations from healthcare providers [51,62]. These reported disparities have been associated with inadequate provider engagement, recommendation practices rooted in perceptions and personal judgement rather than guidelines, as well as provider discomfort discussing sex-related topics [32,63,64]. Providers should prioritize addressing and removing these structural barriers for patients. Additionally, providers in medical specialties that commonly treat females (e.g., pediatricians, OB/GYNs, family practitioners) will serve women well by routinely offering high quality HPV vaccine recommendations [65]. Eliminating missed opportunities to vaccinate is crucial for medically underserved women given their reduced access to and utilization of healthcare services [66]. Increasing recommendation rates during a wide range of medical visits (e.g., annual check-ups, college vaccination, etc.) ensures that recommendations are made wherever and whenever women present in the medical system. The utility of increased recommendation rates can be illustrated through the observation that study participants were vaccinated via a variety of healthcare providers and appointment types. Vaccine reminders, recommendations during routine screenings, and the use of standing orders may also serve to promote the initiation and completion of the HPV vaccine series [51,67].

Findings from the current study also suggest that a lack of maternal support impedes or delays HPV vaccination. This finding is especially salient for vaccine-eligible adolescents given that the decision to vaccinate against HPV is primarily decided by parents [68]. A lack of support may speak to conservative religious or family cultures in which parents, especially mothers, forego vaccinating their younger daughters [69,70]. Given that some women may consider family attitudes about HPV vaccination [36], the absence of maternal support also has implications for adults who may opt to forgo vaccination. Healthcare providers should take family values into consideration when recommending the vaccine. Study findings also indicate that providers should be prepared to address growing vaccine hesitancy among healthcare professionals [71] when discussing the HPV vaccine with parents working in the healthcare field.

Surprisingly, none of the study participants directly discussed vaccine safety as either a motivator or barrier to vaccinate against HPV. However, this may be due to the fact that some unvaccinated women were not aware of the vaccine. When recommending the HPV vaccine, healthcare providers should be prepared to address safety concerns with vaccine-eligible women as well as mothers of children and adolescents. While we did not interview the mothers of participants who reported a lack of maternal support to vaccinate, it is possible that this lack of support was rooted in concerns around the vaccine’s safety. Prior research has cited safety concerns as the primary reason caregivers of adolescents do not initiate the HPV vaccine series [72].

The themes uncovered in this study reveal the need to disseminate vaccine messages that address multi-level factors to vaccination and speak to not only individual knowledge but also to HPV risk perceptions, empowerment, protecting one’s health, family support, and trusted healthcare provider recommendations. The study’s findings have implications for adapting the EBCCP for the Planned Parenthood setting to reach women attending these health centers for preventive and reproductive healthcare. In a subsequent study [73], eight prototypical vaccine decision narratives from the current study were translated into four scripts. The scripts were then delivered via a health kiosk in the PPSP waiting room and pilot tested with young adult women attending this health center.

Limitations of this qualitative study include that it was conducted in a single U.S. geographical area and safety net health center (PPSP). Women’s experiences and views attending this particular health center may differ from women attending health centers in other parts of the country. While not generalizable, these findings may be transferable and applicable for improving HPV vaccine uptake among similar populations. A second limitation may include recall bias as participants were asked to reflect on their past HPV vaccine decision making process and prior conversations with healthcare providers. Additionally, social desirability bias may have affected responses. Social desirability can be problematic when heterogeneity in responses is diminished. To minimize social desirability bias, we enrolled both vaccinated and unvaccinated women to discover a range of responses. We also implemented a range of techniques such as training interviewers to build rapport with women and hold open, yet private discussions with participants (e.g., emphasizing that there were no right or wrong answers). We also utilized interviewers that were the same race/ethnicity, age, and gender as participants to promote comfort in women authentically disclosing and sharing their experiences. Furthermore, given the time allotted for each interview and the fact that women were enrolled from a health center waiting room, an in-depth interview process was in some cases limited. Lastly, the study was conducted in 2015. While the themes appear to be consistent with past and present literature, it is possible that additional motivators and barriers to HPV vaccination exist, especially in the context of the COVID-19 pandemic.

## 5. Conclusions

The current study adds to a limited body of literature on the HPV vaccine decision making process among medically underserved women utilizing a safety net health center. Communities of color remain relatively isolated with respect to awareness of available reproductive preventative measures such as HPV vaccination [74]. Healthcare providers at safety net health centers are uniquely positioned to deliver influential vaccine recommendations and empower socially vulnerable women to vaccinate against HPV. It is important for healthcare providers to include explicit and routine HPV vaccine recommendations during clinical visits of all types with adult patients and parents of adolescent children. Providers are tasked with the need to identify and address varying disease and vaccine risk perceptions as well as a lack of maternal support. Additional research is needed to not only identify additional multi-level motivators and barriers to vaccination, but to determine how to leverage this information to create effective and empowering HPV vaccine messages that resonate with medically underserved women. Future research is also needed to examine the role of the COVID-19 pandemic on missed opportunities to vaccinate against HPV and the HPV vaccine decision-making process.

## Figures and Tables

**Table 1:** Description of Participant Sociodemographic Information (N = 24).

Sociodemographic Information	Total
Age, M (SD)	22.4 (4.0)
Race/ethnicity, *n* (%)	
African American	18 (75.0%)
Caucasian	2 (8.33%)
Latina	2 (8.33%)
Mixed race/ethnicity	2 (8.33%)
HPV vaccination status, *n* (%)	
Vaccinated *	15 (62.5%)
Unvaccinated	9 (37.5%)
HPV vaccine series completion status, *n* (%)	
Complete	11 (73.3%)
Incomplete	4 (26.7%)
HPV vaccination age among, *n* (%)	
≥18 years old	8 (53.3%)
<18 years old	7 (46.7%)
Sexual activity status, *n* (%)	
Sexually active	21 (87.5%)
Not sexually active	3 (12.5%)
Condom use during sexual activity, *n* (%)	
Condoms not used	16 (66.7%)
Condoms used	8 (33.3%)
Health insurance status, *n* (%)	
Health insurance	18 (75%)
No health insurance	6 (25%)
HPV positive disease diagnosis, *n* (%)	1 (4.2%)

* Defined as participants who received at least one of the three doses in the HPV vaccine series.

## Data Availability

Transcript data and codebook are available upon request to corresponding author.
